# Fast food diet with CCl4 micro-dose induced hepatic-fibrosis –a novel animal model

**DOI:** 10.1186/1471-230X-14-89

**Published:** 2014-05-10

**Authors:** Tarak K Chheda, Pratibha Shivakumar, Satish Kumar Sadasivan, Harish Chanderasekharan, Yogananda Moolemath, Anup M Oommen, Jagannath R Madanahalli, Venkataranganna V Marikunte

**Affiliations:** 1 Preclinical Development, Connexios Life Sciences Pvt Ltd., Bangalore, India; 2 Systems Biology Group, Connexios Life Sciences Pvt Ltd., Bangalore, India; 3 Drug Discovery Group, Connexios Life Sciences Pvt Ltd., Bangalore, India

## Abstract

**Background:**

Non-alcoholic fatty liver disease (NAFLD) is defined as a spectrum of conditions ranging from hepatocellular steatosis to steatohepatitis and fibrosis, progressing to cirrhosis, which occur in the absence of excessive alcohol use. Several animal models capture aspects of NAFLD but are limited either in their representation of the disease stages or use for development of therapeutics due to the extended periods of time required to develop full histological features.

**Methods:**

Here, we report the development of a novel rat model for NAFLD that addresses some of these limitations. We used a fast food diet (FFD) and a CCl_4_ micro dose (0.5 ml/kg B.wt) for 8 weeks in Wistar rats. Serological analyses, gene expression profiling and liver histology studies were conducted to investigate the development of steatosis, steatohepatitis and fibrosis in the FFD-CCl_4_ model when compared to the individual effects of a FFD or a micro dose of CCl_4_ in rats.

**Results:**

The serum biochemical profile of the FFD-CCl_4_ model showed an increase in liver injury and fibrosis. This was also accompanied by a significant increase in liver triglycerides (TG), inflammation and oxidative stress. Importantly, we observed extensive fibrosis confirmed by: i) increased gene expression of fibrosis markers and, ii) moderate to severe collagen deposition seen as perisinusoidal and bridging fibrosis using H&E, Trichome and Sirius Red staining.

**Conclusions:**

In summary, we find that the FFD-CCl_4_ rat model developed NAFLD histological features including, steatosis, inflammation and fibrosis in 8 weeks showing promise as a model that can be used to develop NAFLD therapeutics and liver anti-fibrotics.

## Background

Non-alcoholic fatty liver disease (NAFLD) is defined by a spectrum of conditions that occur in the absence of excessive alcohol use and range from hepatocellular steatosis to steatohepatitis (NASH) and fibrosis, progressing to cirrhosis [1,2]. The current prevalence of NAFLD in the western world is ~20–30% of the population [3,4] and is expected to increase to >40% by 2030 [5], indicating the growing risk for individuals leading a sedentary lifestyle on a high-fat, high-carbohydrate, calorie-rich diet.

Research efforts in NAFLD have been aimed at understanding disease development, progression and pathophysiology simultaneously facilitating drug discovery. To this end, various animal models of NAFLD have been developed involving genetic and diet manipulation, treatment with toxins as well as combination models [6-19]. Genetic models of NAFLD include AOX null mice [6,7], MAT1A null mice [8], liver-specific NRF1 knockouts [9], liver-specific PTEN knockouts [10] and leptin-deficient obese mice [11,12], which develop NAFLD due to a disruption in the antioxidant mechanism, fatty acid metabolism or triglyceride synthesis/secretion. Diet modulations leading to NAFLD include the methionine and choline deficient diet-fed mice [13] and high fat diet (HFD) mice [14,15] wherein, NAFLD severity varies with diet composition, duration of feeding, species, strain, and gender of animals. Well-known chemical-induced models of NAFLD include carbon tetrachloride (CCl_4_) and thioacetamide-treated mice [16,17]. The main advantages of the well-established CCl_4_ model include convenience and establishment of both mouse and rat models with evidence of fibrosis/cirrhosis across routes of CCl_4_ administration. However, multiple reports have revealed considerable disadvantages in the intraperitoneal, subcutaneous, inhalation and oral routes of CCl_4_ administration, including chronic peritonitis, necrosis at injection site with inconsistent fibrosis, respiratory arrest and higher mortality with inconsistent fibrosis, respectively [19].

Recently, Charlton et al. reported the development of a fast food diet mouse model (FFD) of NASH recapitulating features of the metabolic syndrome and NASH with progressive fibrosis [20]. The FFD comprised of high saturated fats, cholesterol and fructose, and mimicked the metabolic profile in NAFLD including obesity and insulin resistance, along with features of NASH such as increased inflammation, fibrosis, ER stress and lipoapoptosis. The model showed significant physiological similarity to human NASH but took 24 weeks to develop all the histological features [20].

Here, we report the development of a novel combination rat model for NAFLD established by modifying the FFD [20] and administering a micro dose (0.5 ml/kg bwt) of CCl_4_ recapitulating steatosis, steatohepatitis and fibrosis in an accelerated manner (8 weeks). The serum biochemical profile of the FFD-CCl_4_ model showed an increase in liver injury and fibrosis when compared to FFD alone, CCl_4_ alone or the chow diet control animals along with significant increase in liver TG, inflammation and oxidative stress in the FFD-CCl_4_ model. Importantly, gene expression markers of fibrosis were significantly elevated in the FFD-CCl_4_ model when compared to FFD or CCl_4_ alone or the chow diet control animals, which was further confirmed by histological staining using H&E as well as assessment of collagen deposition using Trichome and Sirius red techniques. In summary, we find that the FFD-CCl_4_ rat model, in 8 weeks, developed NAFLD histological features including, steatosis, inflammation and fibrosis.

## Methods

### Compliance with ethical requirements

The study protocol, animal maintenance, and experimental procedures were approved by the Institutional Animal Ethics Committee (IAEC) of Connexios Life Sciences. All institutional and national guidelines for the care and use of laboratory animals were followed. This article does not contain any studies with human subjects.

### Development of animal model

Ten week old *Wistar* rats (Charles River Labs, USA) were used. Animals were housed in groups of three in polypropylene cages and maintained at 23 ± 1°C at 60 ± 10% humidity and 12 hour cycles of light and dark with free access to feed and water (*ad libitum*). Animals were randomly assigned to four groups, consisting 10 animals/group per sex. Group G1 comprised of animals on chow diet, G2 animals were fed a chow diet and received a CCl_4_ micro dose of 0.5 ml/kg B.wt, G3 animals were fed a FFD alone and G4 animals were fed a FFD and received a CCl_4_ micro dose of 0.5 ml/kg B.wt. The FFD consisted of 2 g cholesterol and 0.5 g cholic acid mixed with normal chow diet made up to 100 g to increase calorie content in comparison to the chow diet. Corn oil (5 ml/kg b.wt) was administered through oral gavage once daily to all animals in G3 and G4 whereas G1 and G2 animals were given drinking water. Further, 15 g fructose was mixed in 100 ml drinking water for all G3 and G4 animals. Group G2 and G4 were administered CCl_4_ (assay purity: >98%) at 0.5 ml/kg B.wt by oral gavage after dissolving in corn oil once weekly for the first two weeks and then on alternate weeks thereafter (i.e., 4th, 6th and 8th week). Food consumption and body weight were measured weekly for all animals during the experimental period.

The experimental period was 8 weeks and the last CCl_4_ dose was administered 48 h prior to sacrificing the animals. All animals were fasted overnight and body weight was measured. Blood was collected from the orbital sinus under isoflurane anesthesia; serum was separated and subjected for clinical chemistry studies. Animals were sacrificed and necropsied, the liver was excised immediately, weighed and taken to estimate liver triglyceride (TG), glutathione (GSH) and thiobarbituric acid reactive substances (TBARS) and gene expression profile using RT-PCR. The rest was preserved, for histology, in 10% phosphate-buffered formalin.

### Clinical chemistry and biomarker analysis

Serum levels of Aspartate transaminase (AST), Alanine transaminase (ALT), Alkaline phosphatase (ALP), triglyceride (TG), total bilirubin, Gamma-glutamyl transpeptidase (GGT) were measured in automated bio- analyzer *EM360* (Transasia Bio-medicals Ltd) using ERBA Mannheim kits (Transasia Bio-medicals Ltd, India). Serum procollagen type III levels were measured as per manufacturer's instructions using the PIIINP ELISA kit manufactured by USCN Life Science Inc.

### Assessment of liver triglyceride, glutathione and TBARS

100 mg of liver sample was collected in 1 ml PBS (pH 7.4) and lysed using a tissue lyser (25 Hz for 5 min). Liver TG was extracted as per Folch’s method. Briefly, 0.3 ml of 10% liver homogenate was extracted in 1.5 ml of chloroform: methanol (2:1) and the organic layer was dried in a vacuum dryer. The residue was re-suspended in absolute isopropyl alcohol and TG levels were estimated using DiaSys Diagnostic Systems GmbH kit. Levels of total glutathione and TBARS in the liver, known indicators of oxidative stress, were measured as described earlier [21,22].

### Assessment of gene expression profile in liver

Real-time PCR was used to evaluate expression of COL1A1, TIMP1, ACTA2, and TGFβ as markers of liver fibrosis, TNFa and osteopontin were studied for inflammation and FABP4 for fatty acid trafficking. All primers were procured from Integrated DNA Technologies, Germany. Total RNA was extracted from liver using Trizol (Sigma, St. Louis, MO, USA) followed by chloroform extraction and isopropyl alcohol precipitation. cDNA was synthesized by reverse transcription (ABI, CA, USA) and amplified using MESA Green PCR Master Mix (Eurogenetic, Belgium). The Primers sequence for assessment of gene expression profiles are mentioned in Table 1.

**Table 1:** Primers sequence for assessment of gene expression profiles

**Gene**	**Sequence**
TGFb1	**Forward**- TGTGTCCAGGCTCCAAATGT
	**Reverse**- AAGGACCTCGGTTGGAAGTG
COL1A1	**Forward**-TCTCAAGATGGTGGCCGTTA
	**Reverse**-ATCTGCTGGCTCAGGCTCTT
TIMP1	**Forward**-GGCTCTGAGAAGGGCTACCA
	**Reverse**-TCGAGACCCCAAGGTATTGC
Acta2	**Forward**- GTCCCAGTTGGTGATGATGC
	**Reverse**- GGGCCAAAAGGACAGCTATG
TNFa	**Forward**- GAAACACACGAGACGCTGAA
	**Reverse**- CAGTCTGGGAAGCTCTGAGG
Osteopontin	**Forward**-CCTGACCCATCTCAGAAGCA
	**Reverse**-GTGGTCATGGCTTTCATTGG
FABP4	**Forward**-GGCTTCGCCACCAGGAAAG
	**Reverse**-TTCCACGCCCAGTTTGAAGGA

### Pathology, staging of fibrosis

Formalin-fixed liver samples were paraffin-embedded, sectioned at 5 μm and stained using hematoxylin and eosin (H&E) to examine morphology. Masson’s Trichome and Sirius Red staining techniques were used to assess fibrosis. All slides were examined under light microscopy at low (X10), high (X40) magnification and also at X20. Histological staging were conducted by modification of earlier methods [23,24]. Grading and scoring for fatty change, hepatocellular ballooning and inflammation was conducted, by a pathologist, Dr. Harish Chandrasekharan, who carried out blind-fold evaluation to the study, as described in (Additional file 1: Table S1 and Additional file 2: Table S2).

### Statistics

Data are presented as mean ± SEM. Comparisons among groups were performed with one-way ANOVA followed by Dunnett's multiple comparison post-hoc test to identify significant differences between groups, p < 0.05 was considered significant.

## Results

### Animals on FFD with a micro dose of CCl_4_ (0.5 ml/Kg B.wt, po) showed liver injury and fibrosis in 8 weeks

Livers from all animals on FFD with/without CCl_4_ appeared pale, enlarged and showed a significant increase in liver weight (p < 0.001) (Table 2). There were no changes in liver weight and gross morphology in animals that were administered CCl_4_ on chow diet. Across all groups, animals did not show any signs of toxicity or overt behavioral changes. All animals on the FFD with/ without CCl_4_ animals gained >10% weight compared to the chow diet-fed animals without any change in weekly food consumption (Additional file 3: Table S3 and Additional file 4: Table S4).

**Table 2:** Effect on liver oxidative stress markers and liver triglyceride levels

**Parameters**	**Chow diet control**	**0.5 ml**/**Kg B.wt CCl**_**4**_	**FFD**	**FFD +** **0.5 ml**/**kg B.wt CCl**_**4**_
Liver TG (mg/g tissue)	6.32 ± 0.79	8.73 ± 1.27	17.17 ± 0.96***	15.49 ± 1.51***
Liver GSH (μg/mg protein)	7.36 ± 0.62	6.81 ± 0.42	6.95 ± 0.45	2.58 ± 1.10**
Liver TBARS (μM/mg protein)	0.56 ± 0.06	0.60 ± 0.064	3.73 ± 0.67***	2.10 ± 0.29**
Relative Liver weight (g)	2.49 ± 0.04	2.86 ± 0.08	4.77 ± 0.15***	4.85 ± 0.16***

Data are expressed as mean ± SEM. Statistical significance was determined using one-way ANOVA followed by Dunnett’s multiple comparison post test, **p < 0.01 and ***p < 0.001.

To estimate liver injury, we compared the serum biochemical profiles of the FFD-CCl_4_ animals to the FFD, CCl_4_ and chow diet controls. ALT, AST, GGT and ALP (known serum markers of liver injury) and procollagen type III (known serum marker of fibrosis) were significantly elevated in the FFD-CCl_4_ animals compared to the chow diet controls (Table 3). Serum triglyceride levels were reduced significantly in FFD with/ without CCl_4_. Apart from the increase in serum triglycerides, animals on a chow diet administered a CCl_4_ micro dose did not show a significant change in the serum biochemical profile when compared to the chow diet controls. However, we did not observe any changes in blood glucose levels under both fasting and fed condition across all the groups (data not shown). Taken together, these data indicate that the FFD-CCL_4_ rats show an increase in markers of liver injury and fibrosis.

**Table 3:** Serum biochemical profile

**Parameters**	**Chow diet control**	**0.5 ml**/**Kg B.wt CCl**_**4**_	**FFD only**	**FFD +** **0.5 ml**/**kg B.wt CCl**_**4**_
AST (IU/L)	118.0 ± 6.93	126.3 ± 2.85	138.4 ± 6.91*	146.7 ± 6.91**
ALT (IU/L)	54.92 ± 1.25	59.17 ± 0.95	62.73 ± 7.72	69.60 ± 4.15*
ALP (IU/L)	87.67 ± 6.86	102.4 ± 7.44	228.8 ± 38.44***	227.8 ± 31.62***
GGT (IUL)	0.09 ± 0.04	0.06 ± 0.06	0.00 ± 0.00	3.30 ± 1.17***
Procollagen type III (ng/ml)	5.61 ± 0.61	5.65 ± 0.65	8.29 ± 0.58	14.96 ± 2.19***
Triglycerides (mg/dL)	145.3 ± 14.28	96.72 ± 8.54*	77.17 ± 7.90**	75.43 ± 4.30**

Effect on serum clinical chemistry markers; data are expressed as mean ± SEM. Statistical significance was determined using one-way ANOVA followed by Dunnett’s multiple comparison post test, *p < 0.05, **p < 0.01 and ***p < 0.001.

In agreement with the liver injury reflected in the serum biochemical profile, liver TG content was significantly elevated in FFD and FFD-CCl_4_ animals (Table 2). Reactive oxygen species (ROS)-mediated oxidative stress markers were measured to access liver damage. We observed a significant (p < 0.01) depletion in liver glutathione levels in FFD-CCL_4_ animals compared to FFD and chow diet controls. In line with this, liver TBARS was elevated in both FFD and FFD-CCl_4_ animals (Table 2). Animals on the chow diet did not show a significant change in oxidative stress markers with/without CCl_4_. Thus, along with an increase in liver injury, the FFD-CCl_4_ animals also showed an overall increase in liver steatosis and oxidative stress.

### Pro-fibrotic and pro-inflammatory pathways are activated in the FFD-CCl_4_ animals

To follow up on elevated liver injury and fibrosis markers observed in the FFD-CCl_4_ sera, we studied gene expression of key genes in inflammation, fibrosis and fatty acid trafficking known to be involved in NAFLD. The gene expression levels of FABP4 (protein associated with fatty acid uptake, transport and metabolism, implicated in development of NASH) were significantly increased in the FFD-CCl_4_ animals compared to the FFD, CCl_4_ and chow diet controls (Table 2). Similarly, osteopontin, a marker for immune cell activation was significantly increased in the FFD and FFD-CCl_4_ animals compared to the control animals. In agreement with this, TGFβ, a well-established marker of fibrosis and inflammation, was expressed at significantly higher levels in FFD-CCl_4_ animals compared to the FFD, CCl_4_ and chow diet controls. Other markers of extracellular matrix (ECM) deposition including COL1A1 and TIMP1 also showed a significant increase in the FFD-CCl_4_ animals compared to the FFD and chow diet controls (Table 4). ACTA2 (ECM deposition marker smooth muscle actin) did not show significant gene expression changes in FFD, CCl_4_ or the FFD-CCl_4_ animals (Table 4). TNFα (proinflammatory cytokine implicated in fibrogenesis) increased both in the FFD and FFD-CCl_4_ animals. Animals that were administered a CCl_4_ micro dose on the chow diet did not show a significant change in the expression of key genes in inflammation, fibrosis and fatty acid trafficking when compared to the chow diet controls. Similarly, except for the increase in TNFα, the FFD animals also did not show a change in the gene expression profile when compared to the chow diet controls.

**Table 4:** **Gene expression profiles for Fibrosis**, **inflammation and fatty acid trafficking**

***Gene***	**Gene expression profile**			
	**Chow diet**	**0.5 ml**/**Kg B.wt CCl**_**4**_	**FFD**	**FFD +** **0.5 ml**/**kg B.wt CCl**_**4**_
***Fibrosis***				
TGFβ1	1.02 ± 0.09	1.00 ± 0.05	1.22 ± 0.17	1.69 ± 0.14*
COL1A1	1.08 ± 0.17	1.56 ± 0.21	2.25 ± 0.32	13.05 ± 1.88***
TIMP1	1.00 ± 0.05	0.81 ± 0.04	1.74 ± 0.15	2.41 ± 0.42**
ACTA2	1.08 ± 0.08	1.00 ± 0.05	1.01 ± 0.12	0.87 ± 0.12
***Inflammation***				
TNFα	1.05 ± 0.13	0.62 ± 0.04	3.59 ± 0.75**	1.30 ± 0.23*
Osetopontin	1.10 ± 0.34	0.86 ± 0.21	5.43 ± 1.14	9.79 ± 2.43**
***Fatty acid trafficking***				
FABP4	1.05 ± 0.15	1.59 ± 0.28	5.63 ± 0.57	45.46 ± 8.64***

Gene expression TGFB1, COlA1, TIMP1, Acta2, TNFa, FABP4 levels in liver; all data are expressed as mean ± SEM. The data measured by quantitative real-time RT-PCR was statistically analyzed for significant using one-way ANOVA followed by Dunnett’s multiple comparison post test, *p < 0.05, **p < 0.01 and ***p < 0.001.

Together, these data show that the FFD-CCl_4_ animals show pro-fibrotic and pro-inflammatory changes in gene expression which are not shown by FFD or the CCl_4_-treated animals.

### The FFD-CCl_4_ mice show an increase in steatosis, hepatocellular ballooning and fibrosis

**Figure 1:**
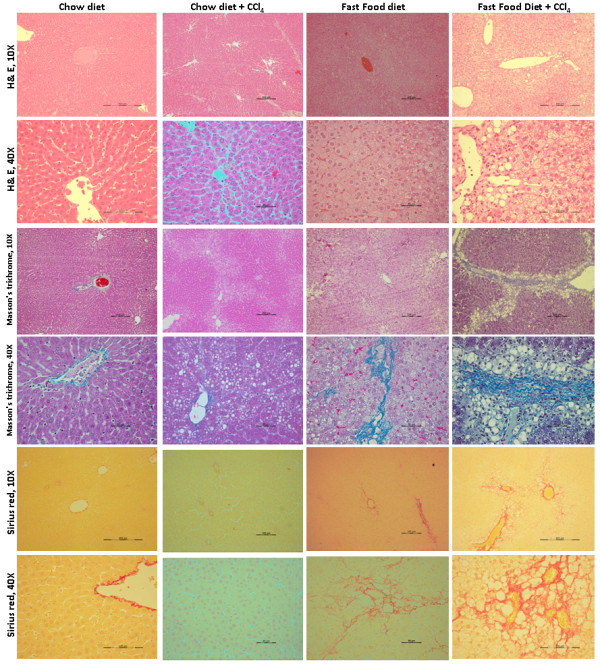
**Hematoxylin-Eosin (H&E, 1st and 2nd rows), Masson’s trichrome (3rd and 4th rows) and Sirius red (5th and 6th rows) – stained sections of liver tissues from chow diet, chow diet + 0.5 ml/Kg B.wt CCl**_**4, **_**fast food diet (FFD), FFD + 0.5 ml/Kg B.wt CCl**_**4 **_**fed animals on 8th week.** There was no steatosis, hepatocellular ballooning or fibrosis in animals fed with chow diet. Mild micro-vesicular fatty changes and mild hepatocellular ballooning, without fibrosis observed in animals fed with chow diet + 0.5 ml/Kg B.wt CCl_4_. Fast food fed animals without CCl_4_ showing moderate micro-vesicular and macro-vesicular fatty changes, hepatocellular ballooning and minimal perisinusoidal fibrosis. FFD + 0.5 ml/Kg B.wt CCl_4_ fed animals showing severe micro-vesicular and macro-vesicular fatty changes with significant hepatocellular ballooning and prominent perisinusoidal, pericellular region with extensive distribution and bridging fibrosis. All liver sections were evaluated at X10 (low-magnification) and X40 (high-magnification).

**Figure 2:**
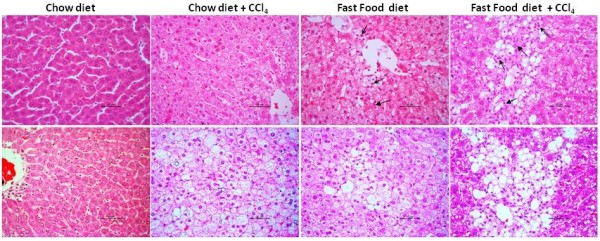
**Row1: Hematoxylin-Eosin (H&E) stained sections of liver tissues from chow diet, chow diet + 0.5 ml/Kg B.wt CCl**_**4, **_**fast food diet (FFD), FFD + 0.5 ml/Kg B.wt CCl**_**4 **_**fed animals on 8th week.** Liver section showing moderate infiltration of inflammatory cells (shown by black arrows) in fast food fed animals with and without CCl_4_ along with moderate micro-vesicular and macro-vesicular fatty changes and hepatocellular ballooning. Mild inflammation was observed in animals fed with chow diet + 0.5 ml/Kg B.wt CCl_4_. Row2: Hematoxylin-Eosin (H&E) stained sections of liver tissues. Liver sections showing increased hepatocellular ballooning in fast food fed animals with and without CCl_4_ along with moderate micro-vesicular and macro-vesicular fatty changes. All liver sections are represented at X40 (high-magnification).

Histological examination of liver sections from FFD-CCl_4_ animals showed a statistically significant increase in micro vesicular and macro vesicular steatosis, hepatocellular ballooning and fibrosis compared to the FFD, CCl_4_ and chow diet controls (Table 5 and Figure 1). As per the scoring system shown in (Additional file 2: Table S2), we observed moderate infiltration of mononuclear inflammatory cells with predominance of macrophages in both the FFD and FFD-CCl_4_ animals along with both periportal and perivenular steatosis and hepatocellular ballooning which are features of steatohepatitis (Figure 2). To evaluate collagen distribution, Masson trichome and picrosirius red staining were carried out (Table 5 and Figure 1). However, liver sections from FFD and FFD-CCl_4_ animals showed increased collagen deposition with the latter showing moderate-severe collagen deposition detectable as perisinusoidal fibrosis and bridging fibrosis (Figure 1). Importantly, this was true for all the animals on the FFD-CCl_4_ regime. Animals on a chow diet with/without CCl_4_ did not show a significant increase in macro vesicular steatosis, hepatocellular ballooning and infiltration of inflammatory cells (Figure 2).

**Table 5:** **Scores of liver from chow diet**, **FFD and FFD**-**CCl**_**4**_

**Histological scores**	**Chow diet control**	**0.5 ml**/**Kg B.wt CCl**_**4**_	**FFD**	**FFD +** **0.5 ml**/**kg B.wt CCl**_**4**_
Fibrosis	00 ± 00	00 ± 00	0.92 ± 0.12***	2.30 ± 0.21***
Steatosis – micro vesicular	0.33 ± 0.14	0.75 ± 0.13	2.00 ± 0.21***	2.60 ± 0.16***
Steatosis – macro vesicular	00 ± 00	00 ± 00	1.92 ± 0.19***	2.50 ± 0.17***
Hepatocellular ballooning	00 ± 00	0.25 ± 0.13	1.00 ± 0.17***	1.50 ± 0.17***
Inflammation	0.75 ± 0.22	1.00 ± 0.25	1.75 ± 0.22**	2.00 ± 0.26**

All data are expressed as mean ± SEM. The data was statistically analyzed for significant using one-way ANOVA followed by Dunnett’s multiple comparison post test, **p < 0.01 and ***p < 0.001.

Hepatocellular ballooning, which has been challenging to establish in animal models of NAFLD, was observed in the FFD-CCl_4_ and the FFD animals but not the CCl_4_-treated animals (Figure 2). Together these data indicate that the FFD-CCL_4_ model recapitulates the steatosis, inflammatory and fibrotic lesions associated with NAFLD.

## Discussion

NAFLD is a spectrum of disorders ranging from fatty liver (steatosis), NASH and fibrosis resulting in cirrhosis [25,26]. Several animal models have been developed to study NAFLD pathogenesis and screen for therapeutics. These animal models vary by way of nature of pathology and evolution of fibrosis [6-19]. Animal models commonly used to study NAFLD include the high fat diet-induced, FFD mouse and CCl_4_-induced liver injury models [16,17,27-29]. The FFD mouse model develops features of human NAFLD. However, it takes about 6 months to establish histological features [20]. Acute/ chronic exposure to CCl_4_ has shown elevated serum liver enzymes, steatosis, centrilobular necrosis, increased liver weight and fibrosis/cirrhosis. However the CCL_4_-induced fibrosis model is very severe and associated with peritonitis, necrosis and lack of consistent development of fibrosis [19]. Chronic CCl_4_ treatment (>2 weeks), on the other hand, is known to invoke adaptive mechanisms, reducing vulnerability to oxidative stress and hepatocellular damage with restorative macrophages showing potential to reverse fibrosis upon CCl_4_ withdrawal [28,30,31].

In the current study, we present a rat model of NAFLD developed over 8 weeks on a modified FFD with a CCL_4_ micro dose (0.5 ml CCl_4_/kg bwt) that captures steatosis, inflammation and fibrosis stages of NAFLD. We used CCl_4_ micro dosing to induce oxidative stress and inflammation without causing overt hepatotoxic effects. In course of our studies, we had tested a micro dose of 1 ml/kg bwt CCl_4_ (unpublished data). However, this resulted in mortality leading us to reduce the dose to 0.5 ml/kg bwt. In this study, we modified the published FFD composition [20] by increasing the amount of fructose used in drinking water to mimic a metabolic overload [32-34] and accelerate disease progression. As expected, all FFD-CCl_4_ animals showed an increase in liver injury, fibrosis and oxidative stress confirmed by changes in serum AST, ALT, GGT, ALP; Procollagen III, and liver GSH and TBARS [35-37], respectively. This was consistent with previous models of CCl_4_-induced fibrosis and the FFD mouse model [20,31,38,39].

Serum triglyceride levels were reduced significantly in FFD with/ without CCl_4_. This decrease is a distinctive feature of CCl_4_ which rapidly increases the triglyceride accumulation in the liver due to a failure in their secretory mechanisms [31,39] and also increased uptake of triglycerides into the liver. We observed, in 8 weeks, an increase in liver TG, fatty acid trafficking, inflammation and fibrosis, which was in consistence with earlier findings where the increase in gene expression of FABP4 (marker of fatty acid trafficking), osteopontin (marker of inflammation), COL1A1 and TIMP1 (markers of fibrosis) [40,41], which have been reported and duration for appearance of liver fibrosis was 24 weeks. In the current study, the liver histological lesions showed all the features of NAFLD in animals treated with FFD-CCl_4_. Development of steatosis is largely due to increased rate of import or synthesis of fatty acids by hepatocytes that exceeds the rate of export or catabolism [42-44]. Steatosis thus developed has an inflammatory response which may be precipitated by a variety of stimuli such as oxidative stress and pro-inflammatory cytokine mediated hepatocyte injury progressing to NASH [43-46].

Considering how this model is distinct from previous models of NAFLD is important and we find that the FFD-CCl_4_ rat model recreates in 8 weeks most histological lesions seen in the FFD mouse [20,47,48] with the exception of a change in serum glycemic/lipid profiles. This model has not shown features of metabolic syndrome thus this model does not exactly mimic human NAFLD. The FFD mouse established by Charlton et al. [20], showed features of metabolic syndrome with hepatocellular ballooning and progressive fibrosis which makes it a good model that mimics human NAFLD but it takes more than 6 months. In the FFD-CCl_4_ model, although we were unable to detect insulin resistance or hyprinsulinemia, we observed steatosis, inflammation and fibrosis associated with NAFLD in 8 weeks.

A significant aspect of this model is that it is able to replicate hepatocellular ballooning and fibrosis in 8 weeks. Developing a rapid fibrosis model in short duration require a variety of stimuli, thus animals fed a high-fat diet for more than 24 weeks are associated with their susceptibility to diet-induced obesity that develop steatohepatitis. In the current model, FFD along with micro dose CCl_4_ provoke an array of responses that results in hepatocellular ballooning, inflammation, and fibrosis. Hepatocellular ballooning has been difficult to establish but was shown recently in the FFD mouse by Charlton et al. [20]. In our study, Masson trichome and picrosirius red staining techniques revealed increased collagen deposition in the form of pericellular and bridging fibrosis. The necroinflammatory foci showed mononuclear infiltration with predominance of macrophages. Further, expression levels of pro-fibrotic and pro-inflammatory gene, TGFβ1 increased along with COL1A1 mRNA levels. Curiously, ACTA2 (ECM deposition marker smooth muscle actin) did not show significant gene expression changes in FFD, CCl_4_ or the FFD-CCl_4_ animals and needs to be further assessed for changes in protein expression.

Interestingly, during model development, we observed a gender bias for NAFLD development with females being more susceptible than males. Further studies will be required to understand the mechanistic reasons for this susceptibility seen in females and also to precisely understand the rate of progression of NAFLD in this model. For example, assessing steatosis, inflammation and fibrosis across 8 weeks at regular intervals will allow for a more accurate interpretation of when these histological lesions develop. This could then be potentially used to identify candidate biomarkers for progression of disease from steatosis to steatohepatitis to fibrosis.

## Conclusions

In summary, we present a fatty liver-induced model of hepatic fibrosis, which captures steatosis, inflammation and fibrosis seen in NAFLD. This model holds promise as a tool for screening for NAFLD therapeutics including liver anti-fibrotics.

## Abbreviations

ACTA2: Alpha-actin-2; ALP: Alkaline phosphatase; ALT: Alanine transaminase; AST: Aspartate transaminase; AOX: Acyl-CoA oxidase; CCl4: Carbon Tetrachloride; COL1A1: Collagen, type I, alpha 1; ECM: Extracellular matrix; FABP4: Fatty acid binding protein 4; FFD: fast food diet; GGT: Gamma-glutamyl transpeptidase; MAT1A: Methionine adenosyltransferase 1A; NAFLD: Non-alcoholic fatty liver disease; PIIINP: Procollagen III N-Terminal Propeptide; PTEN: Phosphatase and tensin homolog; SEM: Standard error mean; TBARS: Thiobarbituric acid reactive substances; TG: Triglyceride; TGFβ: Transforming growth factor beta; TIMP1: Tissue inhibitor of metalloproteinase 1; TNFα: Tumor necrosis factor alpha.

## Competing interests

Tarak K Chheda, Pratibha Shivakumar, Satish Kumar Sadasivan, Vijayragav Dasarahalli Nagaraju, Harish Chanderasekharan, Yogananda Moolemath, Anup Mammen Oommen, Jagannath R Madanahalli, Venkataranganna V Marikunte declare that they have no conflict of interest.

## Authors’ contributions

YM, AMO, VVM designed the research; TKC, PS, SKS, HC, performed the research; YM, VVM, JRM wrote the paper.

## Pre-publication history

The pre-publication history for this paper can be accessed here:

http://www.biomedcentral.com/1471-230X/14/89/prepub

## Supplementary Material

Additional file 1: Table S1 Scheme used for histological staging of fibrosis modified from Kleiner et al., [23]. Click here for file

Additional file 2: Table S2 Scheme used for histological scoring, modified Kleiner et al., [23] and Kawasaki T et al., [24]. Click here for file

Additional file 3: Table S3 Body weight data of chow diet, CCL_4_, FFD and FFD-CCl_4_. All data are expressed as mean ± SEM. The data was statistically analyzed for significant using one-way ANOVA followed by Dunnett’s multiple comparison post test. Click here for file

Additional file 4: Table S4 Feed consumption data of chow diet, CCL_4_, FFD and FFD-CCl_4_. Click here for file
