# Supplementary material for: Fast food diet with CCl4 micro-dose induced hepatic-fibrosis –a novel animal model
Source: BMC Gastroenterol. 2014 May 10;14:89. doi: 10.1186/1471-230X-14-89 (PMC4036109; doi:10.1186/1471-230X-14-89)
Supplement: Additional file 1: Table S1 — Scheme used for histological staging of fibrosis modified from Kleiner et al., [23]. [file 1471-230X-14-89-S1.doc]

**Additional file 1: Scheme used for histological staging of fibrosis modified from Kleiner et al., 2005 (32)**

| **Staging** | **Fibrosis** | **Degree of severity** | **Percentage Fibrosis*** |
| --- | --- | --- | --- |
| 1 | Minimal | Perisinusoidal fibrosis | <10 % |
| 2 | Mild | Lesion easily identified but of limited severity in perivenular, perisinusoidal, or pericellular region with focal distribution | 11 -25 % |
| 3 | Moderate | Fibrosis prominent in perivenular, perisinusoidal, or pericellular region with extensive distribution and bridging fibrosis. | 26 -60% |
| 4 | Severe | Fibrosis complete with periportal fibrosis, prominent bridging fibrosis and cirrhosis. | 61-100% |

*Fibrosis compared to the total tissue area in 25 microscopic fields in each slide
