# Supplementary material for: Fast food diet with CCl4 micro-dose induced hepatic-fibrosis –a novel animal model
Source: BMC Gastroenterol. 2014 May 10;14:89. doi: 10.1186/1471-230X-14-89 (PMC4036109; doi:10.1186/1471-230X-14-89)
Supplement: Additional file 2: Table S2 — Scheme used for histological scoring, modified Kleiner et al., [23] and Kawasaki T et al., [24]. [file 1471-230X-14-89-S2.doc]

**Additional file 2: Scheme used for histological scoring, modified Kleiner et al., 2005 (32) and Kawasaki T et al., 2009 (33)**

| **Score** | **Grade** | **Degree of severity**  **(Score per 25 microscopic fields)** |
| --- | --- | --- |
| **Steatosis** | | |
| 1 | Mild | Up to 33% hepatocytes |
| 2 | Moderate | 33-66% hepatocytes |
| 3 | Severe | > 66% hepatocytes |
| **Hepatocellular ballooning** | | |
| 1 | Minimal | <10 % hepatocytes |
| 2 | Mild | 11 -25 % hepatocytes |
| 3 | Moderate | 26 -50% hepatocytes |
| 4 | Severe | >51 hepatocytes |
| **Inflammation** | | |
| 1 | Mild | < 2 foci per HPF* |
| 2 | Moderate | 2-4 foci per HPF* |
| 3 | Severe | > 4 foci per HPF* |

HPF = High Power field (X40) (as per our scheme)

*At (X20) as per Kleiner et al., 2005 (32)
