# Supplementary material for: Fast food diet with CCl4 micro-dose induced hepatic-fibrosis –a novel animal model
Source: BMC Gastroenterol. 2014 May 10;14:89. doi: 10.1186/1471-230X-14-89 (PMC4036109; doi:10.1186/1471-230X-14-89)
Supplement: Additional file 3: Table S3 — Body weight data of chow diet, CCL4, FFD and FFD-CCl4. All data are expressed as mean ± SEM. The data was statistically analyzed for significant using one-way ANOVA followed by Dunnett’s multiple comparison post test. [file 1471-230X-14-89-S3.doc]

**Additional file 3: Body weight data of chow diet, CCL_4_, FFD and FFD-CCl_4_.**

All data are expressed as mean ± SEM. The data was statistically analyzed for significant using one-way ANOVA followed by Dunnett’s multiple comparison post test.

| **Parameter**s | **Chow diet control** | **0.5ml/Kg B.wt CCl_4_** | **FFD only** | **FFD + 0.5ml/kg B.wt CCl_4_** |
| --- | --- | --- | --- | --- |
| Initial | 238 ± 11.40 | 236 ± 4.25 | 236 ± 10.60 | 235 ± 12.10 |
| Week 2 | 265 ± 13.27 | 254 ± 3.21 | 263 ± 13.30 | 262 ± 8.97 |
| Week 4 | 269 ± 11.70 | 261 ± 3.14 | 275 ± 12.26 | 270 ± 12.85 |
| Week 6 | 272 ± 11.26 | 269 ± 3.69 | 284 ± 11.59 | 280 ± 13.87 |
| Week 8 | 277 ± 8.95 | 266 ± 4.27 | 307 ± 11.77 | 305 ± 10.19 |
