# Supplementary material for: Fast food diet with CCl4 micro-dose induced hepatic-fibrosis –a novel animal model
Source: BMC Gastroenterol. 2014 May 10;14:89. doi: 10.1186/1471-230X-14-89 (PMC4036109; doi:10.1186/1471-230X-14-89)
Supplement: Additional file 4: Table S4 — Feed consumption data of chow diet, CCL4, FFD and FFD-CCl4. [file 1471-230X-14-89-S4.doc]

**Additional file 4: Feed consumption data of chow diet, CCL_4_, FFD and FFD-CCl_4_.**

***All data are expressed as mean ± SEM. The data was statistically analyzed for significant using one-way ANOVA followed by Dunnett’s multiple comparison post test.***

| **Parameter**s | **Chow diet control** | **0.5ml/Kg B.wt CCl_4_** | **FFD only** | **FFD + 0.5ml/kg B.wt CCl_4_** |
| --- | --- | --- | --- | --- |
| Week 2 | 16.31 ± 1.56 | 14.31 ± 0.51 | 13.14 ± 1.04 | 14.30 ± 1.85 |
| Week 4 | 17.00 ± 0.56 | 14.10 ± 0.75 | 15.44 ± 1.75 | 14.97 ± 0.95 |
| Week 6 | 15.97 ± 2.05 | 15.14 ± 0.58 | 14.94 ± 1.43 | 14.08 ± 1.37 |
| Week 8 | 17.72 ± 0.66 | 16.46 ± 0.43 | 13.61 ± 2.08 | 14.36 ± 1.32 |
